# Screen-printed sensors for efficient potentiometric analysis of tolperisone hydrochloride in presence of its co-formulated drugs

**DOI:** 10.1186/s13065-022-00883-1

**Published:** 2022-11-07

**Authors:** Mohamed Rizk, Emad M. Hussein, Safaa Toubar, Emad Ramzy, Marwa I. Helmy

**Affiliations:** 1 Analytical Chemistry Department, Faculty of Pharmacy, Helwan University, EinHelwan, Cairo, 11795 Egypt; 2 National Organization for Drug Control & Research (NODCAR), P.O. Box 29, Cairo, Egypt

**Keywords:** Screen printed, Ion selective electrode, Graphene nano platelets, Tolperisone HCL, Pharmaceutical analysis

## Abstract

Researchers seeking for green chemistry to help safeguard and boost the economy and the environment by discovering unique ways to decrease waste and find substitutes for dangerous chemicals. In this study, a green potentiometric ion-selective electrode (ISE) was developed for measurement of tolperisone HCl (TOLP) in bulk and Pharmaceutical dosage forms in presence of diclofenac sodium and paracetamol as co-formulated drugs. This paper presents the manufacture and characterization of a disposable potentiometric ion-selective strip with an enhanced detection limit for (TOLP) measurement in its tablet dosage form either alone or in presence of the co-formulated drugs. Numerous ion pairs (IPs), such as TOLP-tetraphenylborate (TOLP-TPB), TOLP-phosphotungstic acid (TOLP-PTA), and TOLP-ammonium Reinecke (TOLP- RKT) are tested in presence of different plasticizers. The optimal potentiometric response with a near Nernstian slope of 55.949 mV/decade was achieved within a linear concentration range of 5 $\times$ 10^–5 ^− 1 $\times$ 10^–2^ M using (PTA) and ortho nitrophenyl octyl ether (o-NPOE) as a plasticizer. The effect of the nanoparticles on the membrane stability was studied using the graphene nanoplatelets which have an effective role in the enhancement of some constructed sensors stability. Finally, the developed technique is validated for the estimation of TOLP with high accuracy and precision.

**Graphical Abstract:**

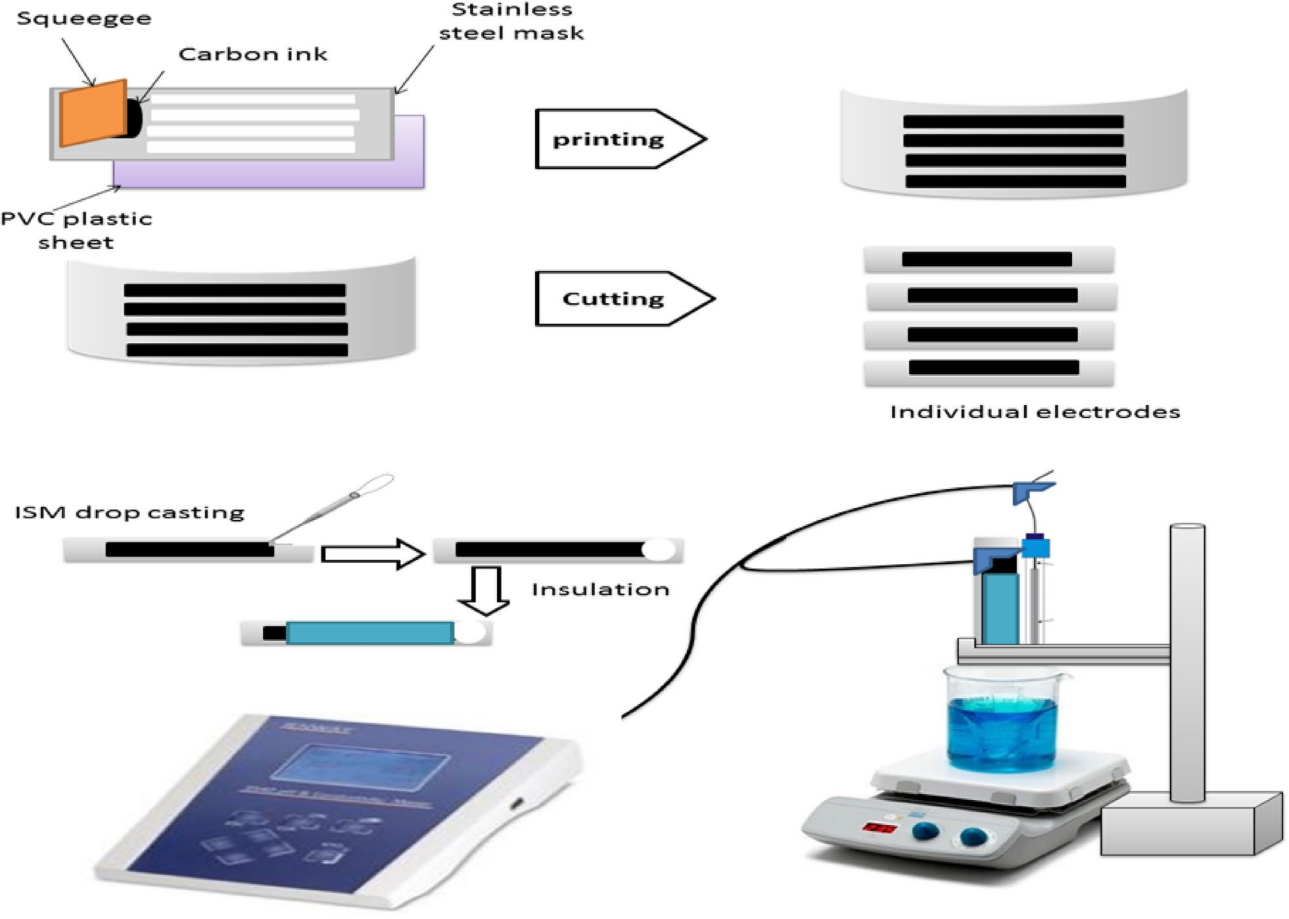

## Introduction

**Fig.1:**
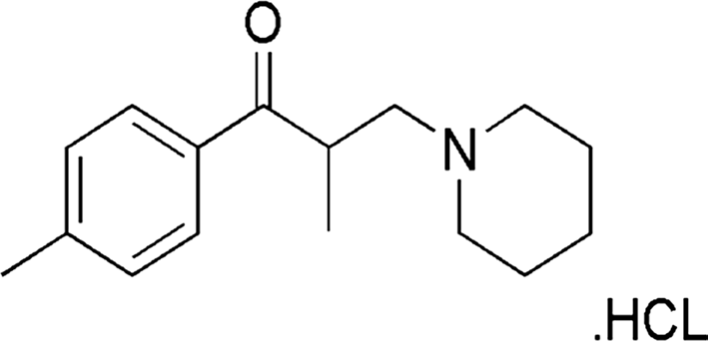
Chemical structure of TOLP

Tolperisone (TOLP) which is chemically designated as 2-Methyl- 1-(4-methylphenyl)-3-piperidin-1-ylpropan-1-one hydrochloride (Fig. 1), belongs to the centrally acting muscle relaxant group and it is prescribed for symptomatic treatment of spasticity, muscle spasm, and osteoarthritis[1]. The reason behind using TOLP in the management of osteoarthritis is suggested to be due to the myokines (released with muscle contraction during function) interaction with structures such as synovial tissue, cartilage, and bone at a molecular level. Two possible pathways by which skeletal muscles interconnect with neighboring joint structures have been hypothesized, including anti-inflammatory and pro-chondrogenic mechanisms [2]. TOLP should be cautiously prescribed to avoid its potential side effects. Thus, it is worthful to develop reliable and simple techniques for monitoring the concentration of TOLP in its pharmaceutical dosage forms.

Literature review revealed that some analytical techniques such as chromatographic methods [3–6] and UV Spectrophotometry [7, 8] had been reported for estimation of TOLP in individual dosage forms and combination with other drugs. Even though good results were achieved, the proposed techniques are solvent and time-consuming and use costly instruments which are not accessible in most laboratories for routine analysis [4–8]. Potentiometric ion-selective electrode (ISE) is a cost-effective analytical technique that can be employed for performing measurements in colored and turbid solutions [9, 10]. The classical (ISE) consists of a plastic membrane (usually made from polyvinyl chloride (PVC)) interpolated between two solutions. One of the two solutions is called the inner reference solution and it is composed of a constant concentration of the analyte; the other solution which is called the sample solution contains inconstant concentrations of the analyte. The potential of the constructed membrane is measured using two reference electrodes, usually Ag/AgCl reference electrodes. Such strategy has numerous drawbacks: big size, large volume of the sample and special skills for construction [9]. The developments of solid-state ion-selective electrodes represent a possible approach to avoid the complications of the classical ISE as the solid-state ISEs have numerous advantages: simple configuration, small size, and small volume of the sample is required [11, 12]. The ion-selective membrane is directly contacted with the solid transducer surface so there is no need for the inner filling solution. In recent times, mass production of economical, movable, and reliable all-solid-state electrodes for pharmaceutical and environmental analysis has become a subject of interest [13]. Such electrodes have already been utilized for rapid and economic estimation of several pharmaceuticals and food additives [14–16] and biological valuable compounds [17].

This work aims to develop a disposable screen-printed ISE for measuring TOLP in pharmaceutical tablet dosage form alone or in presence of diclofenac sodium and paracetamol as co-formulated drugs. The ion exchanger and plasticizer were carefully chosen to optimize the potentiometric response. The electrode's potentiometric response was determined in accordance with IUPAC guidelines [18]. The developed electrodes were used as potentiometric indicator electrodes for the determination of TOLP in its pharmaceutical dosage forms.

## Experimental

### Reagents and chemicals

Tolperisone HCl pure drug was purchased from Combi-Blocks, USA. MYDOCALM^®^ tablets labeled to contain 150 mg tolperisone HCl manufactured by labatec-pharma SA (Geneva), Switzerland, were obtained from local pharmacy. Paramol^®^ 500 mg tablets labeled to contain 500 mg paracetamol was from (Misr Co for Pharm. Ind.S.A.E., Cairo, Egypt). Voltaren^®^50 tablets labeled to contain 50 mg diclofenac natrium was obtained from (Novartis, Cairo, Egypt). Poly (vinyl chloride) (PVC) of high molecular weight, dibutyl phthalate (DBP), ortho nitrophenyl octyl ether (o-NPOE), and graphene nano-platelets powder hydrophobic were purchased from (Sigma-Aldrich, St. Louis, USA). Phosphotungestic acid (PTA) was purchased from (Fluka, Switzerland). Sodium tetraphenylborate (Na-TPB) was from (oxford lab fine chem, India). Ammonium Reinecke (RKT) and monobasic sodium phosphate were from (Riedel–De Haen AG, Hannover, Germany). Graphite powder (particle size < 50 μm) was purchased from (Merck, Darmstadt, Germany). All the reagents were of analytical grade and the experiments were constructed using double distilled water.

### Apparatus

All potential measurements were performed at 25 °C. A Jenway digital ion analyzer model 3503 (Essex, UK) with Ag/AgCl double junction reference electrode, (Steinhum, Germany) was utilized. For studying the influence of pH on the electrode's response, a pH glass electrode (Jenway, UK) was used. The measurements were performed using a magnetic stirrer, Bandelin Sonorox, (Budapest, Hungaria).

### Preparation of ion-pairs (IPs)

For the preparation of (IPs), 25 mL of 0.01 M solution of PTA, Na-TPB or RKT was added to 25 mL of 0.01 M of TOLP solution. After 10 min of stirring, each solution was filtered using a Whatman filter paper. The precipitate was washed several times using double distilled water and dried at room temperature for two days [12].

### Fabrication of screen-printed electrodes (SPEs)

Using a home-made stainless-steel mask, conductive carbon tracks with diameters of 3.0 $\times$ 28.0 mm were manually produced [12]. For printing the carbon tracks, a transparent PVC plastic sheet with a thickness of 200 µm was utilized as support. Graphite powder and PVC dissolved in cyclohexanone: acetone (1:1) to make up the carbon ink. The ink material was pressed during the printing process through the mask against the PVC sheet. For curing, the electrodes were placed in the oven at 60 °C for 3 h. After that, the ion-selective membrane was printed on the graphite carbon track by drop casting. The ISEs were dried at least for 4 h at room temperature. An insulating tape was used to define the sensitive and connecting terminals (3.0 $\times$ 3.0 mm each).

### Calibration graph

The constructed SPEs were conditioned in 0.01 M TOLP for 30 min before measurements then they were submerged with the double junction Ag/AgCl reference electrode in working solutions of TOLP in the range of 1 $\times$ 10^–7 ^− 1 $\times$ 10^–2^ M, the working solutions were prepared in 0.02 M phosphate buffer adjusted to pH (4.50 ± 0.05). Between readings, the constructed SPEs were rinsed with double distilled water and all measurements were done under constant stirring. The emfs as a function of the drug concentrations were recorded, and then the calibration curves of the measured potentials vs. log drug concentrations were drawn.

### Selectivity coefficient of the electrode

The separate-solution method (SSM) was utilized to calculate the electrode's selectivity coefficients [19]. The cell potential of an analyte solution (E_i_) and an interfering ion solution (E_j_) of the same concentration (a_i_ = a_j_ = 1 $\times$ 10^–2^ M) was measured using this approach. The selectivity coefficient (log K^pot.^_i,j_) was calculated according to Nicolsky-Eisenman equation:

$${\text{Log K}}^{{{\text{pot}}}}_{{\text{i,j}}} = \left( {{\text{E}}_{{\text{j}}} {\text{ - E}}_{{\text{i}}} } \right){{{\text{z}}_{{\text{i}}} {\text{F}}} \mathord{\left/ {\vphantom {{{\text{z}}_{{\text{i}}} {\text{F}}} {2.303\,{\text{RT}}}}} \right. \kern-\nulldelimiterspace} {2.303\,{\text{RT}}}} + \left( {1 - \left( {{{{\text{z}}_{{\text{i}}} } \mathord{\left/ {\vphantom {{{\text{z}}_{{\text{i}}} } {{\text{z}}_{{\text{j}}} }}} \right. \kern-\nulldelimiterspace} {{\text{z}}_{{\text{j}}} }}} \right)} \right){\text{Log a}}_{{\text{i}}}$$

where: E_i_ and E_j_ are the potentials of primary and interfering ions, respectively,

R is the gas constant, T is the temperature, z_i_ and z_j_ are the charges of the primary and interferent ions, respectively, and F is the faraday constant.

### Direct potentiometric assay of TOLP in pharmaceutical dosage forms

Ten MYDOCALM^®^ tablets each labeled to contain 150 mg TOLP were accurately weighed individually and the average weight of the ten tablets was calculated, then the ten tablets were transferred to a clean and dry mortar for their conversion to a fine powder. After that; an accurately weighed amount of the resulted fine powder equivalent to 14.1 mg was accurately transferred to 50 mL volumetric flask that contains 30 mL phosphate buffer (pH 4.50 ± 0.05) and sonicated for 15 min using the ultrasonic bath at 25 °C, then the solution was completed to the mark with the same solvent and filtered using 0.45 μm disposable syringe filter. This solution is claimed to have a concentration of 1$\times$ 10^–3^ M TOLP. The selected sensor was used in combination with the Ag/AgCl reference electrode to perform the potentiometric measurements and the concentration was calculated from the corresponding regression equation.

#### Analysis of synthetic mixtures

The Potentiometric measurements were performed for various synthetic mixtures containing a constant concentration of TOLP (1 $\times$ 10^–3^ M) with variable concentrations of co-formulated drugs (diclofenac sodium or paracetamol) in phosphate buffer (pH 4.50 ± 0.05). Using the selected sensor, the potential of each combination was recorded, and the concentration of TOLP was estimated using the associated regression equation.

## Results and discussion

### Effect of membrane composition

Several aspects, including the kind of IP and the kind of plasticizer, influence the construction of polymeric membrane ISEs with an optimal potentiometric response. The IP is essential for thermodynamic ion exchange equilibrium to be established at the membrane/solution interface. As a result, it should be hydrophobic, essentially insoluble in water, and have reasonable stability over a wide pH range.

**Fig. 2:**
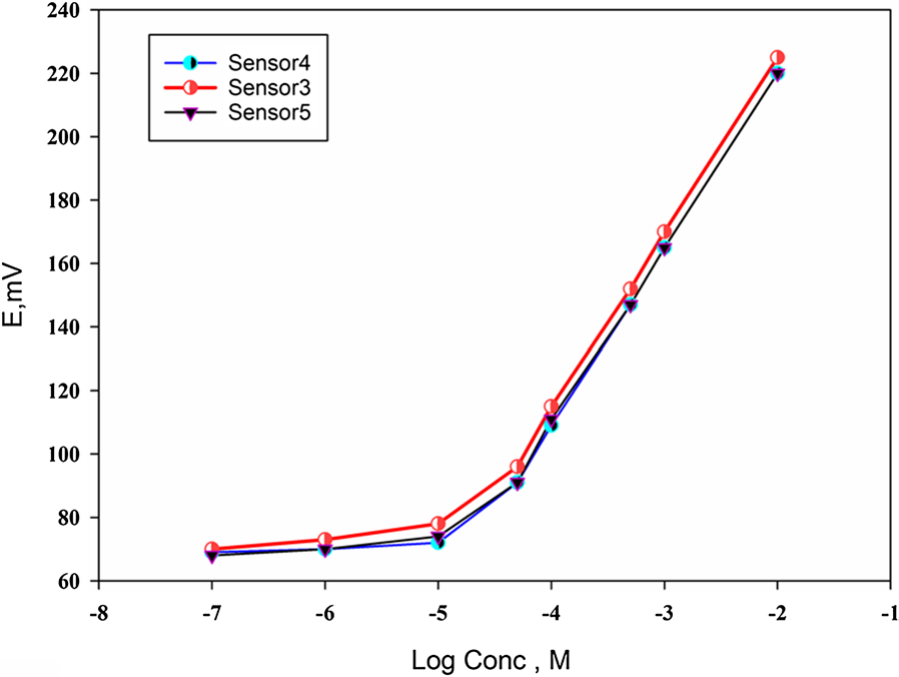
Calibration graphs for TOLP- selective SPE sensors with variable amounts of TOLP-PTA ion exchanger. O-NPOE was utilized as a plasticizer

In–the—meantime, the immiscibility of the plasticizer in water is a must, it should be nonvolatile, and can dissolve the IP. TOLP-PTA as an ion exchanger and DBP as a plasticizer were used for the construction of (Sensor 1), this sensor gave a sub-Nernstian response of 51 mV/concentration decade but unfortunately, this response was not reproducible. So, a thin layer of graphene nanoparticles was introduced under the casted membrane as a trial to overcome this problem. Since the graphene nanoparticles have intrinsic hydrophobicity and electric conductivity, moreover, it gives high potential stability [20] and this enhanced the reproducibility significantly and the slope increased from 51 to 53.019 mV/concentration decade (Sensor 2). Also, we had attempted to enhance the resulted slope by using a different plasticizer which is o-NPOE. Upon using this plasticizer the slope reached to 55.741 mV/concentration decade (Sensor3) and the sensitivity was enhanced which reached to 5 $\times$ 10^–5^ M instead of 1 $\times$ 10^–4^ M (Sensor1,2), this may be due to the higher dielectric constant of o-NPOE (ɛ _o-NPOE_ = 24) [21] and the higher solubility of TOLP-PTA in this plasticizer in comparison with the DBP as it was noticed that mixing TOLP-PTA with DBP gave a turbid solution. Therefore, different amounts of TOLP-PTA were used to determine the optimal amount of the TOLP-PTA ion exchanger and to examine the constructed electrode's robustness. For each membrane composition, the calibration graph, the slope, and the linear range were determined. Over the concentration range of 5 $\times$ 10^–5 ^− 1 $\times$ 10^–2^ M, the constructed sensors (Sensor 3 to 5) showed nearly small changes in slope (55.741 ± 0.209) mV/ concentration decade. The slope was almost unaffected by the minor variation in the amount of TOLP-PTA demonstrating that the constructed electrode has reasonable robustness and the small variation in the quantity of TOLP-PTA in the membrane has little effect on the electrode's potentiometric properties as shown in Fig. 2. Other IPs including TOLP-TPB and TOLP-RNK were also investigated and both sensors displayed slope of 29.5 (Sensor 6) and 50.5 (Sensor 8) mV/concentration decade, respectively using DBP. When using o-NPOE as a plasticizer (Sensor 7), a slope of 29.9 mv/concentration decade was obtained, which is like the slope of Sensor 6. The dramatic decrease in the resulted slope of TOLP-TPB with both plasticizers may be attributed to the leaching of TOLP-TPB from the membrane upon contacting the electrode with the solution. So, Sensor 4 was used for further characterization. All the above-obtained results are summarized in Table 1.

**Table 1:** The characteristics of Potentiometric response of TOLP SPE with variable constituents of the sensing layer

Sensor	% Composition			Slope, mV/concentration decade	Linear range (M)	R^2^
	IP	Plasticizer	PVC			
TOLP-PTA						
Sensor 1	4.8%	63.5% (DBP)	31.7%	51	1 $\times$ 10^–4 ^− 1 $\times$ 10^–2^	0.9897
Sensor 2	4.8%	63.5% (DBP)	31.7%	53.019	1 $\times$ 10^–4 ^− 1 $\times$ 10^–2^	0.9993
Sensor 3	4.8%	63.5% (o-NPOE)	31.7%	55.741	5 $\times$ 10^–5^−1 $\times$ 10^–2^	0.9997
Sensor 4	4.4%	63.7% (o-NPOE)	31.9%	55.949	5 $\times$ 10^–5 ^− 1 $\times$ 10^–2^	0.9998
Sensor 5	5.2%	63.2% (o-NPOE)	31.6%	55.532	5 $\times$ 10^–5 ^− 1 $\times$ 10^–2^	0.9993
TOLP-TPB						
Sensor 6	4.8%	63.5% (DBP)	31.7%	29.5	1 $\times$ 10^–4 ^− 1 $\times$ 10^–2^	0.9991
Sensor 7	4.8%	63.5% (o-NPOE)	31.7%	29.9	1 $\times$ 10^–4 ^− 1 $\times$ 10^–2^	0.9992
TOLP-RNK						
Sensor 8	4.8%	63.5% (DBP)	31.7%	50.5	1 $\times$ 10^–4 ^− 1 $\times$ 10^–2^	0.9992

^*^IP: ion pair

### Effect of pH

**Fig. 3:**
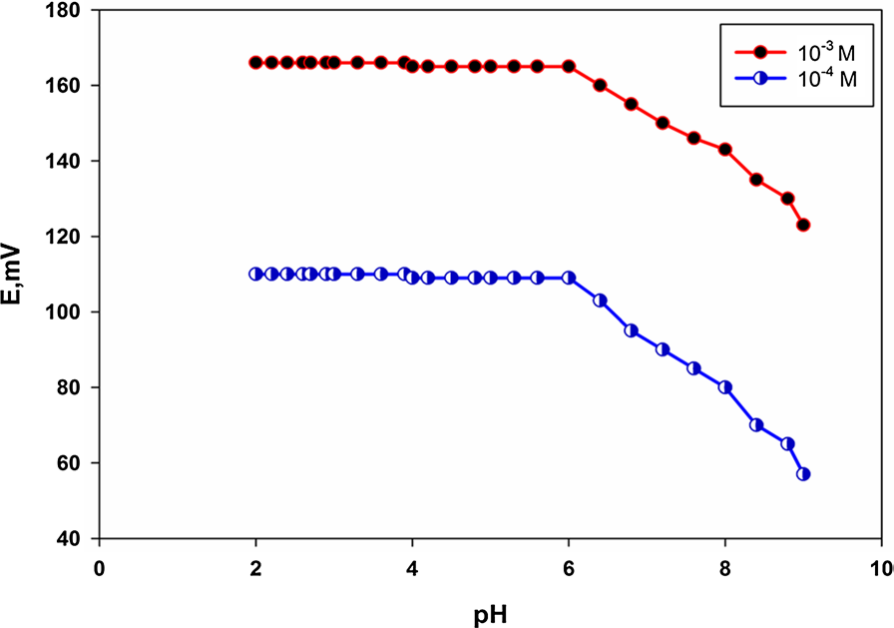
Effect of pH on the response of the selected sensor using **a** 1 $\times$ 10^–4^ and **b** 1 $\times$ 10^–3^ M TOLP solutions

It is very important to measure the electrode potential in the pH range at which the variation in the potential due to variation in TOLP concentration is independent on the pH. So, we studied the effect of the pH on the performance of the selected sensor using two different concentrations 1$\times$10^−4^ and 1$\times$10^−3^ M. The pH of the investigated solution was varied utilizing HCl and/or NaOH solutions. In the pH range from 4 to 6, the emf was unaffected by the pH. At pH˂ 4, there was a very minor shift in emf values, which might be attributable to H^+^ interference. At pH > 6, there was a significant shift in emf values as shown in Fig. 3.So that the measured solutions were prepared in phosphate buffer adjusted to pH (4.50 ± 0.05).

### Response time

**Fig. 4:**
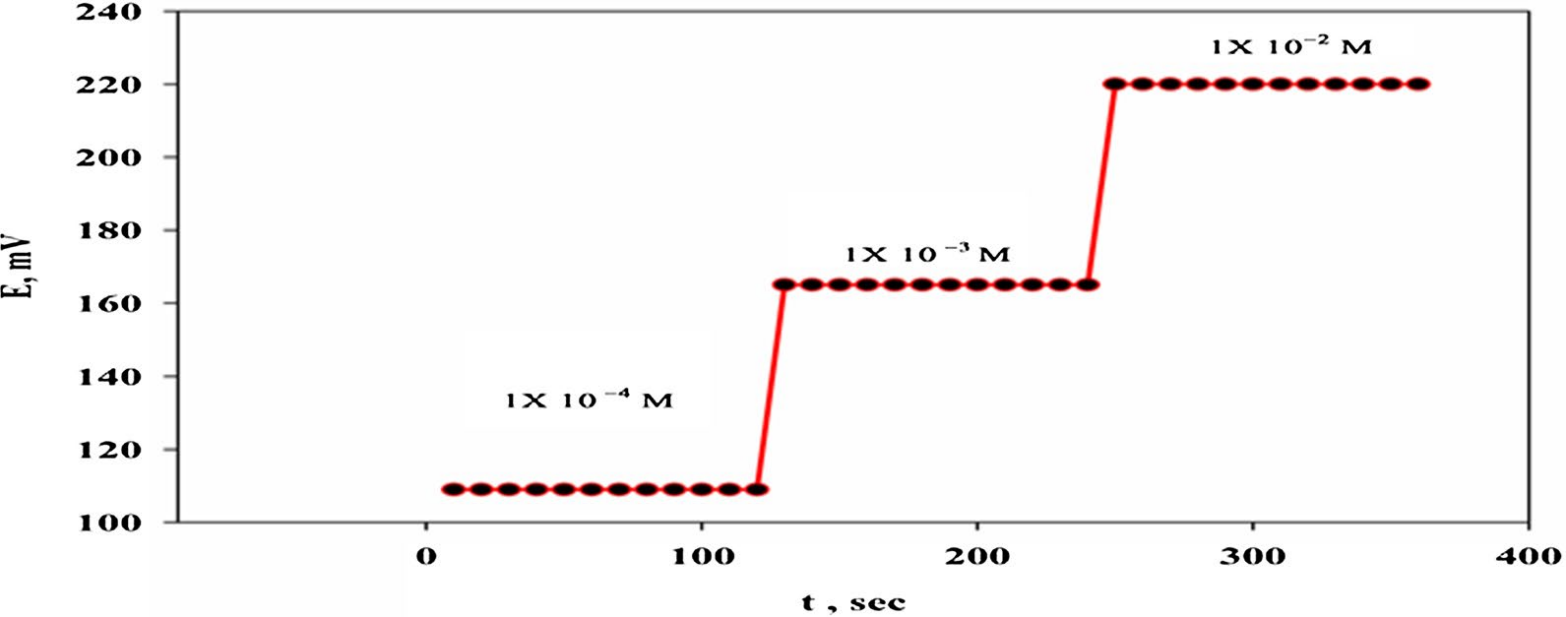
Dynamic response time of the selected sensor at various concentrations

The response time (t_95_) is defined by the IUPAC guideline (1994) [18] as the time the electrochemical cell takes to achieve 95 percent of the steady-state potential after alteration the analyte concentration in the solution. Figure 4 illustrates the dynamic response time of a TOLP-PTA electrode (Sensor 4). The electrode displayed a rapid and stable response upon increasing the concentration of TOLP in the potentiometric cell from 1 $\times$ 10^–4^ to 1 $\times$ 10^–2^ M. The calculated response time (t_95_) was found to be ≤ 10 s.

### Potential drift

**Fig. 5:**
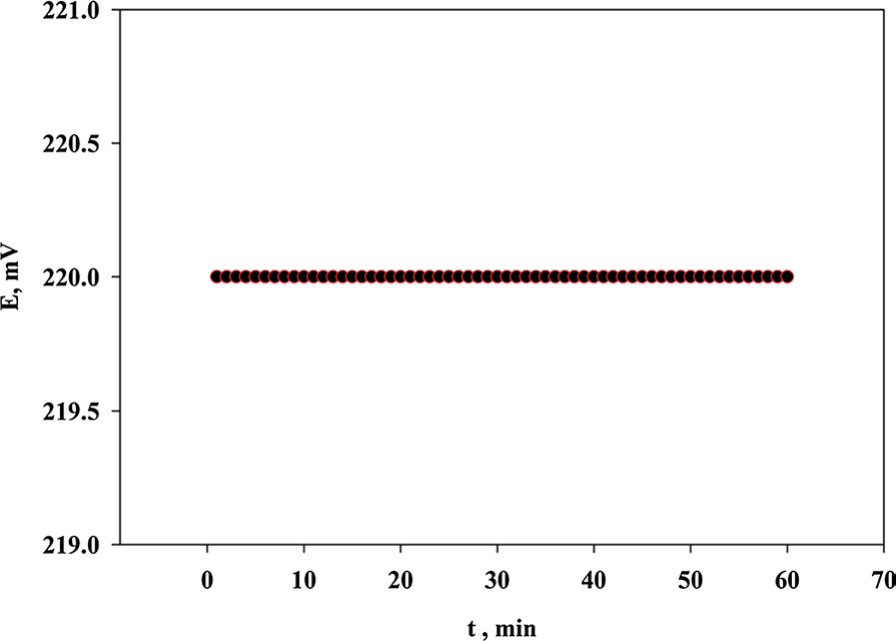
Potential stability of the selected sensor monitored for 60 min at 1 ×10^–2 ^M TOLP solution

For evaluating the potential drift of the selected (Sensor 4), the stability of the electrode potential was assessed by soaking the electrode in a stirred solution of 1 × 10^–2^ M TOLP for about 60 min. The reading was stable, and it was not changed even to a minor change over the investigated time indicating high potential stability of the selected electrode as shown in Fig. 5. The causes of this high stability are the high stability of the IP, the compatibility between the IP and the selected plasticizer, and the inner solid carbon contact is less prone to polarization.

### Hysteresis and reproducibility

**Fig. 6:**
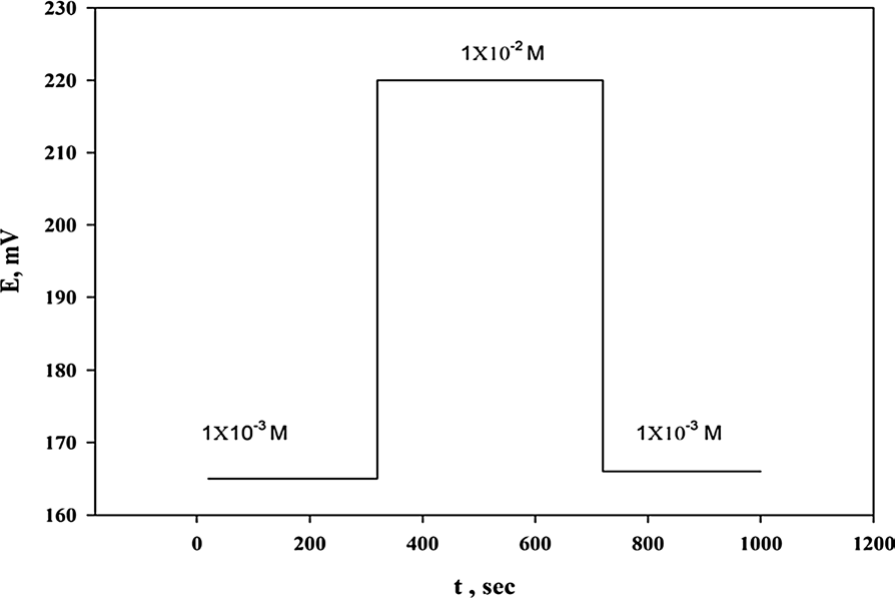
Potential reproducibility of the selected sensor when exposed to various solutions of TOLP

In the potentiometric ISE studies and according to IUPAC [18], hysteresis is defined as the difference in electrode potentials observed for the same analyte solution after exposing the electrode to a variable concentration solution. TOLP–PTA (Sensor 4) hysteresis was investigated by measuring electrode potential in 1 × 10^–3^ M solution for 300 s, then exposing the electrode to 1 × 10^−2^ M solution for 400 s. The electrode was returned to the first solution for 300 s. When the electrode was transferred between TOLP solutions of varying concentrations, a quick and steady potential response was produced. After 400 s of exposure to 1 × 10^–2^ M, the electrode kept its initial potential (+ 1 mV) as shown in Fig. 6. The electrode has little hysteresis and high reproducibility, according to the obtained results.

### Selectivity of the electrode

The capacity of the ISE to discriminate between the target analyte and an interferent is described by the selectivity coefficient (log K^pot.^_i,j_). Using (SSM), the selectivity coefficients of the TOLP sensor to a range of interfering chemicals were determined. As mentioned above, TOLP is formulated alone or combined with diclofenac sodium or paracetamol, so it is very important to investigate the interference of these drugs during the determination of TOLP, as a consequence, the logarithmic values of the selectivity coefficient were calculated using SSM for these two drugs and they were found to be − 3.45 and − 2.77 for diclofenac sodium and paracetamol, respectively as illustrated in Table 2. These small logarithmic values of the selectivity coefficient indicating high selectivity of the electrode to TOLP in presence of these two drugs. When primary and interfering ions are present at the same time in practical applications, the electrode may behave substantially differently than when only one kind of ion is present. However, the results obtained during analysis of synthetic mixtures including varying ratios of the studied drug to co-formulated drug revealed that the chosen sensor may be utilized successfully for selective measurement of TOLP in the presence of either diclofenac sodium or paracetamol as co-formulated drugs as shown in Table 3. Also, the potential interference of common ions and amino acids was investigated; the results revealed that the developed sensor has high selectivity toward TOLP over these interfering compounds as shown in Table 2.

**Table 2:** Selectivity coefficients of the selected SPE electrode via the separate solution method

Interferents	log K^Pot^_primary ion, interferent_
Paracetamol	− 2.77
Diclofenac sodium	− 3.45
L- alanine	− 2.59
glycine	− 2.68
DL glutamate	− 2.41
Na^+^	− 2.52
K^+^	− 2.83
Ca^2+^	− 3.13
L-leucine	− 2.79
Maltose	− 2.81

**Table 3:** Estimation of TOLP in MYDOCALM^®^ 150 mg Tablet and in laboratory prepared mixtures containing various ratios of diclofenac sodium or paracetamol by the selected sensor

%Recovery^a^ of TOLP in MYDOCALM^®^ Tablet	TOLP: diclofenac sodium Ratio	% Recovery^a^ ± SD of TOLP	TOLP: paracetamol Ratio	% Recovery^a^ ± SD of TOLP
100.53 ± 0.93	3: 1	100.73 ± 0.61	1: 2	101.14 ± 0.35
	3:2	100.73 ± 0.61	1: 2.2	100.33 ± 0.35
	3: 4	99.92 ± 0.35	1: 3	99.92 ± 0.70

^a^Average of three determinations

### Validation parameters

The developed technique was validated as stated by the International Conference on Harmonization (ICH) guidelines [22] and the IUPAC recommendation [18].

The developed method's linearity is investigated in triplicates using varied concentrations of TOLP standard solutions. Table 4 summarizes the data and shows that the correlation coefficients are close to unity, indicating that the developed method is linear.

**Table 4:** Response parameters of the selected sensor

Parameters	Value
Slope (mV/ concentration decade)	55.949
Intercept	332.15
Square correlation coefficient (R^2^)	0.9998
Linearity range (M)	5×10^-5^ − 1×10^-2^
LOQ (M)	5 × 10^–5^
LOD (M)	2 × 10^–5^
%Mean recovery (± SD)	100.01 ± 0.33
Relative standard deviation (%RSD)	0.33

**Fig. 7:**
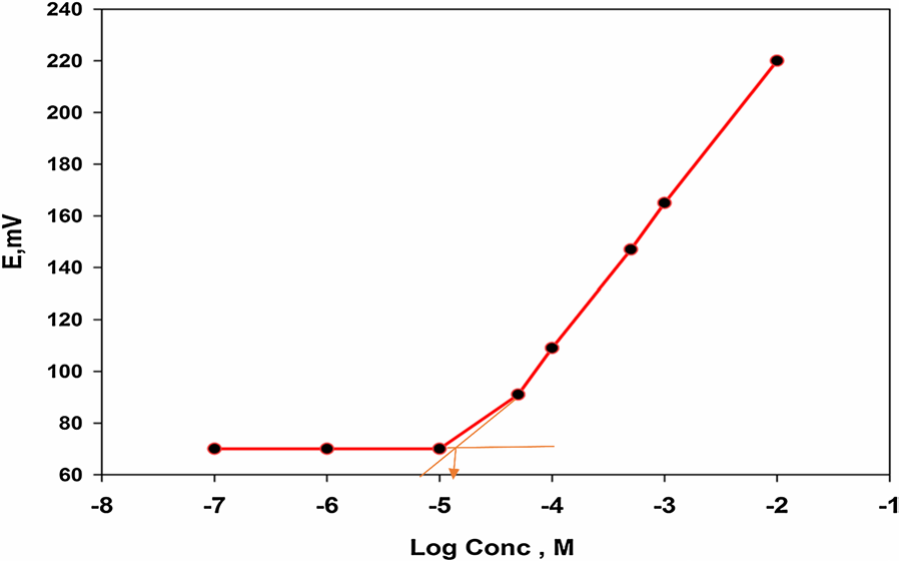
Determination of the detection limit of the selected sensor. The intersection of the two segments of the calibration graph shows the detection limit of the electrode

The selected sensor's limit of detection (LOD) was determined using the IUPAC guideline [18]. The LOD is the concentration at the point where the two calibration plot segments meet as shown in Fig. 7.It was found that the LOD was 2 × 10^−5^ M as shown in Table 4.

The accuracy and precision of the obtained results were verified by determining three distinct concentrations of pure TOLP using the selected sensor by applying the standard addition method. The % recovery and the standard deviation values are computed as shown in Table 5, demonstrating that the suggested approach is accurate and precise.

### Analytical application

The selected sensor was effectively used for the determination of TOLP in tablet dosage form using standard addition technique and the obtained results revealed that the constructed sensor could successfully determine TOLP in its tablet dosage form with excellent recovery as shown in Table 5. Also, the selected sensor was effectively used to determine TOLP in pharmaceutical formulations (MYDOCALM^®^150 mg) as well as synthetic combinations containing diclofenac sodium and paracetamol without treatment, the results showed that excipients in susceptible pharmaceutical formulations did not cause any interference as illustrated in Table 6. The obtained results were statistically compared with that of reported method [6] using Student’s t-test and variance ratio F-test and it was found that there was no significant difference between the performance of the two methods as shown in Table 6.

**Table 5:** Accuracy and precision for estimation of TOLP in the pure drug and MYDOCALM 150 mg tablets

Pure drug					
Taken mg/50 ml	Found	Mean found (mg/50 ml)	Mean %recovery	Mean% recovery	SD
8.46	8.21	8.36	98.82	98.26	0.48
	8.53				
	8.34				
11.28	11.14	11.05	97.96		
	10.98				
	11.02				
14.10	12.97	13.82	98.01		
	14.57				
	13.93				
MYDOCALM 150 mg tablets					
8.46	8.62	8.48	100.24	100.00	1.41
	8.30				
	8.51				
11.28	11.41	11.11	98.49		
	11.02				
	10.90				
14.10	13.62	14.28	101.28		
	14.70				
	14.52

**Table 6:** Estimation of TOLP in its tablet dosage form and in its laboratory prepared mixtures with diclofenac sodium or paracetamol by the developed potentiometric procedure and reported method [6]

Dosage form	MYDOCALM 150 mg	TOLP: diclofenac sodium at ratio 3:1	TOLP: paracetamol at ratio 1:2.2	Reported HPLC method [6]
Mean % recovery^a^ ± SD	99.52 ± 0.61	99.92 ± 0.93	100.33 ± 0.93	98.8 ± 0.98
N	3	3	3	3
Students t test (2.776) at p = 0.05	1.079	1.441	1.959	
F test (19) at p = 0.05	2.644	1.133	1.133	

^a^Average of three determinations

## Conclusion

Green, simple, economic, and a novel method based on using a disposable home-made potentiometric ISE was developed and validated for the determination of TOLP in pure form and in its pharmaceutical dosage forms either alone or combined with diclofenac sodium or paracetamol as co-formulated drugs. Numerous ion exchangers and plasticizers were considered for the fabrication of a TOLP selective electrode aiming to achieve a wide range of linearity and high sensitivity. The optimal results were obtained using PTA as an IP and o-NPOE as a plasticizer, the selected sensor showed a near Nernstian response of 55.949 mV/ concentration decade over the concentration range (5 $\times$ 10^–5^ − 1 $\times$ 10^–2^ M). Also, the electrode potential response was not affected over the pH range (4–6). The potential stability was checked for 60 min in TOLP solution and the results revealed that the selected sensor showed high potential stability. Furthermore, the effect of the nanoparticles on the stability of the developed sensors was studied using graphene nanoplatelets, the results showed that the graphene nanoplatelets have a great effect on the stability of the membrane in case of using DBP as a plasticizer but it has a negligible effect on the potential of the selected sensor. Finally, based on the promising obtained results, this constructed sensor could be utilized for routine analysis in the quality control laboratories for the determination of TOLP in pure form and in its pharmaceutical dosage forms either alone or combined with diclofenac sodium or paracetamol as co-formulated drugs.

## Data Availability

All the data associated with this research has been presented in this paper.

## Abbreviations

ISE: Ion-selective electrode
TOLP: Tolperisone HCl
TOLP-TPB: TOLP-tetraphenylborate
TOLP-PTA: TOLP-phosphotungstic acid
TOLP- RKT: TOLP-ammonium reinecke
o-NPOE: Ortho nitrophenyl octyl ether
PVC: Poly (vinyl chloride)
IP: Ion-pair
SPEs: Screen-printed electrodes
SSM: The separate-solution method
LOD: Limit of detection
LOQ: Limit of quantification and
DBP: Dibutylphathalate
M: Molar
